# The Appropriate Indicator Should be Used to Assess Treatment Failure in STH Infections

**DOI:** 10.4269/ajtmh.2011.11-0317a

**Published:** 2011-09-01

**Authors:** Antonio Montresor, Dirk Engels, Lester Chitsulo, Albis Gabrielli, Marco Albonico, Lorenzo Savioli, Patrick Lammie

**Affiliations:** Department of Control of Neglected Tropical Diseases; World Health Organization; Geneva; E-mail: montresora@who.int

Dear Sir:

We write to comment on the article by Humphries and others^1^ recently published in the *American Journal of Tropical Medicine and Hygiene*: in our opinion, the study is well conducted, however, we respectfully disagree with the conclusions and in particular with the claim made by the authors about the failure of albendazole treatment of hookworms.

We summarize here the reasons of our disagreement:

Humphries and others base the claim for albendazole failure in treating hookworm infection on the analysis of *cure rate* (CR) results that they measured at 61%; however, in the case of infections caused by hookworm and other soil-transmitted helminths (STHs), the CR is not a good indicator of drug efficacy because it is influenced by the intensity of infection at baseline and by the sensitivity of the parasitological method used to recover eggs from stool.^2^ As a result, it is very difficult to compare CRs obtained in different settings and reach meaningful conclusions about differences in drug efficacy. Our opinion is that drug efficacy in STH infections should be assessed with a more apt indicator, namely the egg reduction rate (ERR).

The data presented by Humphries and others show an ERR of over 80% and this corresponds to the normal range of efficacy of albendazole against this parasite,^3^ as correctly reported by the authors.

We would also like to stress the fact that the main objective of preventive chemotherapy against hookworm and other STH infections (those by *Ascaris lumbricoides* and *Trichuris trichiura*) is not to achieve cure of infected individuals, but rather to significantly reduce the number of worms harbored by such individuals^4^; this is because the average observed reduction (that can be measured by counting the number of eggs passed out in feces) is sufficient to cause the parallel decrease:

• of transmission (because of the reduced number of eggs contaminating the environment)^5^; and
• of morbidity (because the few surviving worms—that cannot replicate in the human host—cause less harm to the human host).^6^

As an additional comment, we would also like to express our concern about the method used by the authors to assess drug quality (i.e., *in vitro* anthelminthic activity), because this method has never been validated for albendazole that is insoluble in water; in addition, in our experience, the key factors in determining the quality of drugs used against STH are the tablet dissolution and disintegration times, and none of them was tested by the authors.

Finally, we strongly disagree with the authors' statement that “*mebendazole and pyrantel are not any longer recommended for the treatment of hookworm infection*”; again, the studies on which the authors formulate their conclusion are based on the evaluation of the cure rate,^7^,^8^ and therefore, for the reasons mentioned previously, not conclusive. As a matter of fact, the World Health Organization (WHO) continues to include mebendazole and pyrantel in the list of recommended drugs for the treatment of STH infections.

The WHO is seriously concerned about unjustified claims of drug resistance made on the basis of incorrect interpretations of parasitological results and on the poor understanding of the STH biology and control strategies. These claims create uncertainty among the managers of control programs about the efficacy of the control measures to be implemented and undermine the scaling up of STH control activities.

As a consequence, a working group reporting to the WHO Strategic and Technical Advisory Group on Neglected Tropical Diseases (NTD) was established with the aim of developing a Standard Operating Procedure to be implemented whenever a control manager suspects a reduction of the drug activity (report available at http://www.who.int/neglected_diseases/NTD_STAG_Report_2011.pdf).

Although we hope that this much needed tool will be available before the end of the year, we take this opportunity to invite *AJTMH* and other scientific journals to carefully assess the grounds on which claims of drug failure are based should similar papers be submitted for publication.
